# False Confessions: A Study Space Analysis

**DOI:** 10.1002/bsl.70008

**Published:** 2025-07-17

**Authors:** Laura Farrugia

**Affiliations:** ^1^ Department of Psychology Faculty of Health and Life Sciences Northumbria University Newcastle Upon Tyne UK

**Keywords:** false confessions, interrogation, study space analysis, vulnerability

## Abstract

Confessions are one of the most powerful types of evidence in the criminal justice system. Despite the vast amounts of psychological research conducted, false confessions still remain a pervasive problem around the world. Thus, an examination of the existing work conducted to date is needed to identify any gaps in knowledge or areas of further enquiry. A study space analysis was conducted to explore the adequacy and concentration of studies on false confessions. Using a combination of a number of key terms such as ‘false confessions’, ‘interrogation’, and ‘vulnerable adults’, a search of five databases was conducted. Overall, 230 studies were included in the final analysis. A total of 25 independent variables and 15 dependent variables were identified. However, the study space analysis revealed gaps concerning gender, vulnerability, and training and instructions regarding confessions. A lack of work exploring false confessions across crime types and severity was also discovered.

## 1 Introduction

Individuals still find it difficult to believe that anyone would falsely confess to a crime of which they are completely innocent (Blandon‐Gitlin et al. 2011). However, false confessions are not a new phenomenon; they continue to be a pervasive problem in criminal legal systems around the world (Brimbaletal. 2024; K. Kassin 2014; Scherr et al. 2020). Indeed, statistics show that more than 25% of wrongful conviction cases later exonerated by DNA evidence involved a false confession (Innocence Project 2019). Thus, the prevalence of false confessions purports the need to explore the existing literature base in order to identify gaps in knowledge and areas of further investigation.

The most basic definition of a false confession is when an individual confesses to a crime of which they are completely innocent (G. Gudjonsson 2018). Munsterberg (1908) was one of the first scholars who started exploring false confessions and much of his work threads into current knowledge regarding the distinct types of false confessions, now referred to as voluntary, coerced‐compliant and coerced‐internalised (see S. Kassin and Wrightsman 1985, for a full discussion). Although the Kassin‐Wrightsman classification has been critiqued, most notably by Ofshe and Leo (1997), it still remains one of the most comprehensive explanations to the different types of false confessions.

### 1.1 Why Do False Confessions Occur?

There has been a substantial amount of research conducted into false confessions and why they may occur. Predominately, scholars have reported that false confessions are the product of the interrogation process (Catlin et al. 2023; Drizin and Leo 2004). The Reid Technique (Inbau et al. 2013), used in America and other countries around the world, has been heavily criticised for its association with eliciting false confessions (S. Kassin et al. 2010; Livingston et al. 2019) despite the authors claiming that it elicits confessions only from guilty suspects. Described as an accusatorial method, it is characterised as being guilt presumptive and confession‐oriented and employs a number of psychologically manipulative techniques designed to elicit confessions. Such techniques include physical isolation, minimisation tactics (such as downplaying the seriousness of the crime) or maximisation (emphasising the seriousness of the offence), preventing denials of guilt, and lying to the suspect (S. Kassin et al. 2010). The False Evidence Ploy (Ofshe and Leo 1997), for example, can include the suspect being presented with a murder weapon and being told that their DNA evidence is present on it, or that an eyewitness has placed them at the scene. Such a tactic is used to convince the suspect that they have little option but to confess and that a conviction is inevitable (Ofshe and Leo 1997). The bluff tactic involves deception but introduces a level of uncertainty. For example, interrogators may inform the suspect that there is CCTV of the crime that when reviewed will implicate them and so is used to motivate a confession. Furthermore, interrogations which result in a false confession tend to last longer. This can lead to the suspect experiencing sleep deprivation with subsequent implications for impaired decision‐making (Frenda et al. 2016). This can result in innocent suspects providing a false confession. For example, it is well established that individuals focus on short‐term consequences and often discount the long‐term consequences when making decisions (Kalenscher and Pennartz 2008). If an interrogator suggests to a suspect that making a confession will end the interrogation (a short‐term reward) and the possible criminal charges are minimised (long‐term consequences), the suspect is likely to engage in such temporal discounting to leave the intolerable interrogation environment and come to believe that providing a confession is their only valid method of doing so (Cabell et al. 2020; S. Kassin et al. 2010). Thus, the accusatorial interrogation method and techniques used within it can persuade innocent suspects to falsely confess (Horselenberg et al. 2003; Perillo and Kassin 2011).

### 1.2 Implications of False Confessions

Confessions (whether true or false) are regarded as one of the most powerful sources of evidence (S. Kassin et al. 2010; S. Kassin 2012). Research has continuously documented the strength of confession evidence in leading to conviction at trial (Drizin and Leo 2004; S. Kassin and Neumann 1997). Whilst this does not present a problem if the confession is true, scholars have reported that individuals tend to over believe confessions, even when they are aware that the confession has been obtained under coercion and/or has been contradicted by other powerful evidence, such as DNA evidence (Appleby and Kassin 2016; Kassin and Sukel 1997). What this can lead to is innocent individuals being wrongfully convicted based on a confession that was elicited using coercive and manipulative interrogation techniques (Appleby et al. 2011; S. Kassin et al. 2010; Scherr et al. 2020).

Such belief in false confessions is not limited to the layperson. The presence of a confession can trigger confirmation bias in the legal professionals that are involved in the case. For example, scholars have reported that the mere knowledge of a confession can alter subsequent evidence collection and interpretation (see J. Kukucka and Kassin 2014, e.g.), leading legal professionals to seek, interpret and create new evidence which confirms preexisting beliefs of guilt (S. Kassin et al. 2013). This has also been reported within the interview process. In one study, S. Kassin et al. (2003) reported that participants tended to choose interrogation questions and tactics if they thought the subject was guilty. This has been reported in subsequent studies (Narchet et al. 2011). What this means is that when an individual provides a false confession, they are likely to be ‘treated differently at every subsequent stage of the criminal process’ (R. Leo 1996, 298), with legal professionals less likely to pursue other potential leads due to the confession tainting other forensic evidence. It comes as no surprise that individuals who provide a false confession end up wrongfully convicted (Scherr et al. 2020).

When an innocent individual is convicted based on a false confession, it means that the real perpetrator goes unpunished and free to commit further offences. Researchers have estimated that over 40,000 further crimes per year are committed by perpetrators in cases where there has been a wrongful conviction (Norris et al. 2020). Subsequently, a false confession and wrongful conviction produces two harms—an individual, innocent of any involvement in the crime, is punished and, even if exonerated, will find it difficult to re‐enter society (Kukucka and Evelo 2019), and the true perpetrator remains unpunished and free to commit further offences. This alone warrants further investigation into the work conducted into false confessions.

### 1.3 Existing Research

Given the weight of confession evidence and the increasing numbers of wrongful convictions due to a false confession, researchers have devoted considerable attention to this subject area (S. Kassin et al. 2010). Deslauriers‐Varin (2022) identifies three major waves of research within the scientific literature. The first wave draws on the theoretical understanding of false confessions with research suggesting that individual characteristics, crime‐related factors and the interview/interrogation context could play a significant role in an individual's decision to provide a false confession (Beauregard et al. 2010). The second wave is reported to have focused on validating the theoretical explanations offered in the first wave with the overall consensus that vulnerable individuals are more likely to falsely confess to crimes (Gudjonsson et al., 2004). The third wave, drawing on research spanning the last decade, has focused on situational factors, particularly the effect of interrogation techniques and the context in which interrogation takes place. The perceived strength of evidence, the way the evidence is presented, the manner in which the interrogation is conducted, and the techniques used have all been found to influence whether an individual will confess (L. Brimbal and Luke 2022).

It is clear that a substantial amount of research has been conducted into false confessions in terms of what they are, why they may occur and their implications. However, despite this knowledge, the prevalence rates of false confessions are still considered high by many. For example, the National Registry of Exonerations (2024) estimated that 13% of 3608 exonerations were due to a false confession, although many false confessions are misclassified (Welner et al. 2024). However, given the implications of a false confession on the innocent individual involved, and the overall criminal justice outcomes, it is important to examine the existing research base to systematically determine where (if any) significant gaps in knowledge remain (Purssell and McCrae 2020).

### 1.4 The Current Study

The current study uses a Study Space Analysis technique (SSA; Malpass et al. 2008) to examine the adequacy and concentration of studies on false confessions. The SSA will explore the type of variables manipulated and the types of variables measured. In addition, ecological validity is examined focussing broadly on the type of study, the population type, instruments used and the type and severity of crime context. The representativeness of location is also explored to consider where the plethora of research lies. The SSA methodology does not look at the statistical robustness of the interventions on the outcome, but focuses more on topic coverage (Malpass et al. 2008). It reveals gaps in the literature by creating a visual representation of all of the relevant studies and their variables via the development of matrices. Areas within the matrices that have a low or null frequency counts suggests a lack of research interest (Malpass et al. 2008). This methodology differs from a systematic review and meta‐analysis. The focus of the SSA is on the *variables* that have been examined in order to identify gaps in the literature, whilst the latter two approaches tend to review and analyse the *findings* from the studies included (Anh and Kang 2018).

The SSA methodology has been used within a number of related topics (e.g., see Memon et al. 2010 for their SSA on the Cognitive Interview, and Waterhouse et al., 2020 for their SSA on multiple interviewing of child witnesses). However, to the author's knowledge, the SSA methodology has not been applied to explore the adequacy and concentration of studies on false confessions.

## 2 Method

A study space analysis (SSA) was conducted on existing literature that explores false confessions. The search criteria, data collection and sources, and the procedure is outlined below.

### 2.1 Data Collection

The data for the SSA was obtained via searches of online databases including Google Scholar, PsychINFO, PsychARTICLES, PubMed and Science Direct between January 2024 and December 2024. The search terms included a combination of ‘false confessions’, with ‘juvenile’, ‘vulnerable adults’, ‘interrogation’, ‘mental health’ and ‘intellectual disability’. Overall, the initial search produced 1152 published pieces of work. After irrelevant studies, duplicates and studies not fitting the inclusion criteria were sifted out (in line with other SSA's, see Memon et al. 2010; Waterhouse et al. 2020 e.g.), a total of 183 papers remained that included 230 studies.

### 2.2 Inclusion Criteria

The inclusion criteria for studies included in the current SSA were:

1. The presence of testing, manipulating, surveying or measuring elements that may lead to a false confession and/or some psychological element related to it, for example, suggestibility, compliance or acquiescence.
2. Peer reviewed.
3. Published in English.

The criteria were kept as broad as possible to ensure as many relevant pieces of work that focused on false confessions were included. From the initial 1,152 pieces of work found, 455 duplicates were removed, and 514 were removed given that they did not fit the inclusion criteria, for example, review papers (*n* = 301), irrelevant topic (*n* = 37) etc. The remaining 183 papers were available electronically and included 230 studies for the final analyses.

### 2.3 Procedure and Coding

First, all independent and dependent variables for each study were identified and listed before subsequently being plotted into a matrix. The independent variables were plotted down the left‐hand side and the dependent variables were listed along the top, thus creating one overall IV*DV matrix. Frequency counts for each independent variable against its corresponding dependent variable were entered. Please note, some studies included more than one independent and dependent variable. Next, the ecological validity was explored, for example, whether the study was an experiment, survey or a case analysis in nature, against the participant population types to examine the most commonly occurring types of work against the different participant population samples. Next, all independent and dependent variables were plotted on the left‐hand side against the participant population types creating another matrix. Finally, the location of each study was examined to explore the most commonly occurring location of the research work, the type of methodology and the type of participant population. All materials and data for these analyses are available upon request from the author.

## 3 Results

### 3.1 Independent and Dependant Variables

A total of 25 independent variables were identified in the SSA and related to a range of different areas. The majority of these related to a confession. For example, studies frequently manipulated whether a confession was present (Appleby et al. 2011; Evans and Lyon 2019; Gudjonsson et al., 2009; Volbert et al. 2019), the source of the confession/evidence (Alceste et al. 2019; Bernhard and Miller 2018; DiFava et al. 2023; Marion et al. 2016) and type of confession (Horselenberg et al. 2003; Mindthoff et al. 2020; Pasala 2022; Sigurdsson and Gudjonsson 1996b), the veracity of the confession (Gudjonsson etal. 2016; Redlich et al. 2011; Villar et al. 2014; Wojciechowski et al. 2018) and if training in confessions had been received (Cleary and Warner 2016; Kassin etal. 2007). In addition, the participant or suspect type as well as the type of crime was also manipulated. For example, the age or gender of the participant (Bettens et al. 2024; Candel et al. 2005; Drake et al. 2017; Gudjonsson and Henry 2003) was examined, as was the severity of the crime (Brimbal et al. 2024; Hall et al. 2020). Independent variables relating to the interrogator and interrogation details were also identified. These focused predominately on interrogator characteristics (Shifton 2019), the type and length of the interrogation (Hagsand et al. 2023; Jones and Penrod 2018) and the type of interrogation tactics used (Barnes et al. 2023; Horgan et al. 2012).

A total of 15 dependent variables were also identified in the SSA. These focused on confessor's characteristics (e.g., the characteristics of offenders or exonerees such as interrogation details, number of false confessions and the offence type; Cleary 2014; Feld, 2013; Gudjonsson et al.2021a, 2004b, 2004, 2007a, 2012, 2007b; Willen and Stromwell 2012a) as well as psychometric test scores (Billings et al. 2006; Drake 2010; Drake et al.^2015^, 2015b, 2016; Gudjonsson 1990; Gudjonsson and Sigurdsson^1999, 2010^; Gudjonsson et al. 2021), false confession rates (Blair, 2007; Drake et al. 2016, 2017; Henderson and Levett 2018; Paton et al. 2018), levels of perceived guilt (Larmour et al. 2015; Scherr et al. 2020; Wojciechowski et al. 2018; Woody and Forrest 2009) and confidence in the guilt decision (Kassin and Sukel 1997; Shifton 2019, 2021; Snyder et al. 2009) amongst others. Most studies in the SSA included more than one independent and dependent variable.

Generally, there was a wide spread of independent variables being examined against the dependent variables. The most commonly explored independent variable was the impact of participant type on dependent variables such as the confessor's characteristics (Bettens et al. 2024; Goldstein et al. 2003; Redlich et al. 2010), the psychometric test scores (Arndorfer et al. 2015; Billings et al. 2006; Drake 2010; Drake et al.^2015^, 2015b; Giostra and Vagni 2024; Gudjonsson and Young 2021; Ray and Jones 2012), false confession rates (Candel et al. 2005; Drake et al. 2017), confession knowledge (Alceste et al. 2020b; Costanzo et al. 2010; Henkel et al. 2008) and perceptions of the interrogation (Appleby and McCartin 2019; Hagsand et al. 2022; Redlich et al. 2008). The impact of confession veracity was also frequently examined against confessor's characteristics (Malloy et al. 2014; Redlich et al. 2011), psychometric test scores (Geven et al. 2020; Gudjonsson et al. 2012, 2016; Villar et al. 2013) and perceived guilt (Alceste et al. 2020a; Kassin and Sukel 1997; Levine et al. 2010; Wojciechowski et al. 2018). The presence of a confession and its impact on dependent variables, including confessor's characteristics (Appleby and McCartin 2019; Hagsand et al. 2022; Redlich et al. 2008; Malloy et al. 2014), psychometric test scores (Redlich et al. 2011; Geven et al. 2020; Gudjonssonetal. 2012, 2016; Villar et al. 2013), and perceived guilt (Alceste et al. 2020a; KassinandSukel 1997) and confidence in guilt decision (Levine et al. 2010) tended to be examined frequently too. On the other hand, there were some independent variables that were examined only once or twice against the dependent variable. e.g., the impact of the source of confession/evidence that manipulates where the confession/evidence came from, e.g., self‐report, false incriminating witness was rarely explored in relation to the interviewee characteristics—i.e., the impact of the confession/evidence was rarely examined against the perceived pressure to confess, nor the perceptions of the consequences of confessing. Similarly, the type of confession obtained (e.g., coerced v voluntary) was also rarely examined in relation to interviewee characteristics. Furthermore, the impact of the type of defendant/suspect typically used in vignette studies rarely focused on the gender of the defendant/suspect and was rarely examined against the confidence in the guilt decision. This is also true for the impact of jury instructions on perceived guilt, the presence of the confession on confidence in guilt decision and sentencing perceptions. The independent variable relating to training in confessions was infrequently examined against other dependent variables such as perceived guilt and the confidence in guilt decision (see Table 1).

**TABLE 1:** Overall matrix depicting independent variables (IV's) and dependent variables (DV's) across all populations, locations and methodologies.

	DV's														
IV's	Confessor's characteristics	Interviewee characteristics	Psychometric test scores	Perceived guilt	Confidence in guilt decision	Authenticity of confession	Medium/Format of confession	False confession rates	Account details	Impact of evidence	Perceptions of interrogation	Sentencing perceptions	Interrogator characteristics	Confession knowledge	Truth and deception detection
Confession															
Presence of confession	34	3	65	16	2	8	1	20	9	5	7	2	0	2	0
Source of confession/evidence	0	1	3	37	9	7	0	8	1	17	14	4	0	13	0
Confession contamination warning	0	0	0	4	0	3	0	0	0	1	1	0	1	0	0
Jury instructions	0	0	0	1	0	1	0	0	0	1	1	0	0	0	0
Confession rehearsal	0	0	0	2	1	3	0	0	0	0	0	0	0	1	0
Confession motivation	1	6	5	3	2	1	0	20	1	1	6	1	0	6	0
Confession type	0	0	15	15	5	6	0	7	0	3	5	5	0	0	0
Confession details	0	0	1	7	3	5	0	0	0	2	0	0	0	0	0
Confession veracity	64	10	43	25	4	2	0	3	15	6	20	0	0	2	0
Confession medium	0	2	6	9	5	3	0	8	13	3	4	0	0	1	0
Confession analysis	0	0	0	2	0	0	0	0	3	0	0	0	0	0	0
Perceptions of confessions	0	2	0	2	1	0	0	6	0	2	4	0	0	4	0
Training in confessions	10	0	0	2	2	0	5	4	0	0	5	0	12	0	5
Participant/Suspect/Crime details															
Participant type	240	43	378	27	6	7	0	54	5	16	39	5	6	65	1
Defendant/Suspect type	2	8	0	42	6	26	0	0	0	10	13	8	0	4	0
Suspect status	6	15	6	6	2	3	2	21	3	8	13	0	0	6	1
Crime type/Severity	12	0	9	16	6	9	0	5	2	4	8	4	0	2	0
Interrogator/Interrogation details															
Interrogator characteristics	2	0	0	6	2	2	0	0	0	4	1	0	0	4	0
Interrogator incentive	0	0	0	1	0	0	0	0	0	0	3	0	0	0	0
Interrogation type	3	3	4	16	3	4	2	6	10	6	9	0	1	0	0
Interrogation length	2	5	2	14	6	5	1	4	0	4	8	1	0	4	1
Interrogation content	0	0	0	2	0	4	0	0	2	2	4	0	0	0	0
Interrogation tactics	9	10	21	13	3	3	0	28	10	7	33	4	0	5	1
Evidence type	4	0	10	22	10	8	0	11	0	9	2	1	0	4	0
Data analysis	0	1	3	1	1	0	0	2	0	0	0	0	0	0	0

### 3.2 Ecological Validity

The methodologies used in the research studies included in the SSA were examined. Overall, the research tended to be experimental (*n* = 130; Wojciechowski et al. 2018; Bettens and Redlich 2024; Gudjonssonetal. 2007a, 2009a), or survey driven in nature (*n* = 89; Pearse et al. 1998; Scherr and Normile 2022; Arndorfer et al. 2015; Gilbert et al. 2024). A total of 11 studies focused on analysing real‐life cases or interrogations (Gudjonsson,Sigurdsson,etal. 2006a; Haney‐Caron et al. 2023; Horselenberg et al. 2003). These will be explored in turn.

#### 3.2.1 Experimental Data

The studies included in the SSA used a number of experimental paradigms (*n* = 49). For example, three studies (Hasel and Kassin 2009) used a modified version of the marble game paradigm whereby the participant is tasked with keeping an arbitrary item (a marble) hidden from a confederate who repeatedly questions them about its location, thus designed to simulate a brief interrogation. However, the majority of studies (e.g., Scherr et al. 2020) used either the Alt‐Delete computer crash paradigm (*n* = 12; Hasel and Kassin 2009); participants complete a letter typing exercise and are directed not to press the ‘alt’ key or the computer will crash—however, the programme is designed to crash on the 96^th^ letter; or the cheat test paradigm (e.g., Frenda et al. 2016; *n* = 17; Guyll et al. 2013) whereby participants are instructed to work individually on some problem tasks but are persuaded to ‘cheat’ by assisting a confederate.

The experimental data also included an array of crime contexts as part of their methodology. These tended to use vignettes or case scenarios that drew upon real life cases (*n* = 24). For example, studies used cases such as the Marvin Anderson rape case (*n* = 3; Marion et al. 2016), the Christopher Ochoa murder case (*n* = 3; Serpa 2009) and the confession of Bradley Page (*n* = 2; Alceste, Luke, et al. 2020b). The most commonly real‐life case drawn upon was that of State v Wilson (*n* = 5; Gudjonssonetal. 2000)—a case concerning rape and murder. Other studies used mock crimes (e.g., those not relating to a known case) as the context of their study. These ranged from minor offences such as mock thefts (*n* = 8; Gudjonssonetal. 2004, 2007a) and mock assaults (*n* = 3; Gudjonssonetal. 2007b; North et al. 2008) to more serious offences such as mock robbery (*n* = 8; Volbert et al. 2019; Bettens and Redlich 2024), and mock sexual assault (*n* = 2; Feld 2013). The most commonly used mock offence was murder/manslaughter (*n* = 16; Richardson et al. 2014; Houck et al. 2021).

In the experimental studies, a number of different instruments (*n* = 34) were used to measure participant characteristics. Some instruments were used only once or twice such as the Wechsler Abbreviated Scale of Intelligence (Haney‐Caron et al. 2023) and the Pretrial Juror Attitude Questionnaire (Barnes et al. 2023). Others were drawn upon more frequently including the Gudjonsson Suggestibility Scale (*n* = 8; Haney‐Caron et al. 2023) and the Gudjonsson Compliance Scale (*n* = 6; Horselenberg et al. 2003). Please note, some studies used more than one instrument.

#### 3.2.2 Survey Data

A large number of the survey data tended to draw upon individuals either in custody or incarcerated for a range of crimes (*n* = 27). These varied from minor crimes such as driving or traffic offences (*n* = 10; Bettens et al. 2024; Cleary and Bull, 2021; Zannella et al. 2024; Gudjonsson and Sigurdsson 2010; Snyder et al. 2009; Gudjonsson et al. 2008, 2010) to more serious crimes such as sexual assault (*n* = 10; Deslauriers‐Varin et al. 2011; Gubi‐Kelm et al. 2020; Gudjonsson and Sigurdsson 1999; Holt and Palmer 2023; Malloy et al. 2014) and/or murder/manslaughter (*n* = 6; Goldstein et al. 2003). Quite often, these individuals were incarcerated for more than one crime.

In examining the survey data, a number of different instruments (*n* = 48) were used throughout, with more than one instrument frequently being used. These tended to focus on mental health difficulties such as the Anxiety and Depression Checklist (*n* = 1; Gudjonsson et al. 2008b), the Brief Psychiatric Scale (*n* = 2; Peters et al. 2012), the Hospital Anxiety and Depression Scale (*n* = 1; North et al., 2008) and the Mini International Neuropsychiatric Interview (*n* = 1; Gudjonsson et al. 2010). Other measures focused on the participants' personality characteristics; for example, the Eysenck Personality Questionnaire (*n* = 8; Sigurdsson and Gudjonsson, 1996a, 2001; KassinandSukel 1997), the Minnesota Multiphasic Personality Inventory (*n* = 1; Frumkin et al. 2012), and the NEO Personality Inventory (*n* = 3; Drake, 2010), or the level of participants' intelligence through the use of the Wechsler Abbreviated Scale of Intelligence tests (*n* = 12; Gudjonsson 1990; Pearse et al. 1998). The most commonly used instrument referred to the measurement of suggestibility, compliance and the likelihood to confess through the use of the Gudjonsson Suggestibility Scale I and II (*n* = 26; Peters et al. 2012; Redlich et al. 2003), the Gudjonsson Compliance Scale (*n* = 17; Gudjonsson 1990; Gudjonsson et al. 2021), and the Gudjonsson Confession Questionnaire (including the revised version; *n* = 6; Gudjonsson and Sigurdsson, 1999; Gudjonsson,Sigurdsson,Einarsson,et al. 2010a; Iliya et al. 2022).

#### 3.2.3 Case Analysis

This type of data focused on the analysis of real‐life police interviews/confession statements (*n* = 8; Cleary 2014; Davis et al. 2005; Feld 2013; Hagsand et al. 2022, 2023) or the analysis of cases taken from the National Registry of Exonerations (*n* = 3; Bettens and Redlich, 2024; Gudjonsson,Sigurdsson,et al. 2008b). The offences in these cases tended to be of a more serious nature; for example, sexual offences (*n* = 6; Davis et al. 2005; Hagsand et al. 2023), murder/manslaughter (*n* = 7; Davis et al. 2005; Hagsand et al.^2023^, ^2022^), and robbery (*n* = 3; Feld 2013).

### 3.3 Population Representativeness

The population representativeness refers to the diversity of the participants who took part in the included studies in the SSA. For the purposes of the SSA, this was split into five main participant groups:

- General/Community—this refers to an adult sample drawn from the community, including those who are eligible for jury selection.
- Student—this refers to sample groups drawn from colleges or universities whereby students were over the age of 18 years.
- Child—this includes all children participants (under the age of 18 years) but excluded juvenile offenders.
- Offender—this sample includes all juvenile and adult suspects and offenders, and also includes papers that draw on analysis of real‐life police interviews and cases taken from the National Registry of Exonerations.
- Professional—this sample includes criminal justice professionals such as defence attorneys, psychiatrists, forensic medical examiners and expert witnesses.

Please note, some studies included a mixture of samples, for example, general/community and student populations. Each population group will be explored in turn in terms of independent and dependent variables, and methodology type. See Table 2.

**TABLE 2:** Matrix depicting independent variables (IV's) and dependent variables (DV's) across participant population types in all methodologies.

Participant population type	General/Community population	Student population	Child population	Offender population	Professional population
Independent variables					
Confession					
Presence of confession	9	17	3	13	0
Source of confession/Evidence	16	17	1	0	0
Confession contamination warning	3	1	0	0	0
Jury instructions	1	0	0	0	0
Confession rehearsal	1	1	0	0	0
Confession motivation	7	14	3	2	0
Confession type	6	9	0	1	0
Confession details	2	3	0	0	0
Confession veracity	6	28	0	7	2
Confession medium	2	11	0	0	2
Confession analysis	0	2	0	0	0
Perceptions of confessions	3	4	0	0	0
Training in confessions	0	2	0	0	18
Participant/Suspect/Crime details					
Participant type	34	102	27	98	10
Defendant/Suspect type	7	16	0	1	2
Suspect status	7	14	3	0	2
Crime type/Severity	8	5	0	4	1
Interrogator/Interrogation details					0
Interrogator characteristics	1	1	0	1	2
Interrogator incentive	0	1	0	0	0
Interrogation type	7	13	1	5	1
Interrogation length	4	5	0	2	0
Interrogation content	0	2	0	1	0
Interrogation tactics	13	28	6	6	2
Evidence type	10	13	1	2	1
Data analysis	0	2	0	0	1
Dependent variables					
Confessor's characteristics	4	46	0	76	4
Interviewee characteristics	7	33	6	0	3
Psychometric test scores	35	122	22	78	2
Perceived guilt	47	73	1	0	6
Confidence in guilt decision	12	21	1	0	2
Authenticity of confession	20	23	0	0	1
Medium/Format of confession	1	0	0	0	2
False confession rates	8	73	8	0	4
Account details	0	22	3	7	0
Impact of evidence	14	22	0	2	0
Perceptions of interrogation	32	37	2	9	4
Sentencing perceptions	6	8	1	0	0
Interrogator characteristics	1	1	0	0	0
Confession knowledge	28	26	4	0	3
Truth and deception detection	1	1	0	0	2

#### 3.3.1 General/Community Sample

Overall, a general/community sample was used in 63 studies in the SSA (DiFava et al. 2023; Forrest et al. 2012; Hall et al. 2020; Howard 2019; Pasala 2022; Zannella et al. 2024). The most commonly manipulated independent variables in this sample was the participant type, for example, the gender of the participant, or the race of the participant (*n* = 34; Henkel et al. 2008), the source of the confession/evidence, for example, whether the confession evidence was withheld or disclosed, or whether the evidence was a false confession or eyewitness misidentification (*n* = 16; Henderson and Levett, 2020; Gudjonsson and Sigurdsson 1999; Kukucka and Evelo 2019; Iliya et al. 2022) and the type of interrogation tactics used, e.g., whether the tactic was overt or psychological, or the type of feedback provided by the interrogator (e.g., unfriendly or friendly) (*n* = 13; Hall et al. 2020; O’Connell et al. 2005; Davis et al. 2005). Minimal studies (if any) explored the impact of jury instructions (*n* = 1; Pasala 2022), confession details (*n* = 2; Alceste et al. 2019) confession medium (*n* = 2; Alceste et al. 2020a), perceptions of confessions (*n* = 3; Mindthoff et al. 2018) or interrogation content (*n* = 0) within this sample.

The most commonly used dependent variables within this sample group included perceived guilt, for example, the rate at which participants thought the suspect was guilty (*n* = 47; Scherr et al. 2018; Schneider and Sauerland 2023; Hagsand et al. 2023), psychometric test scores (*n* = 35; Wachi et al. 2018), confession knowledge, such as the perceived reliability of the confession or the likelihood the suspect could go home if they confessed (*n* = 28; Henderson and Levett 2020; Hagsand et al. 2023; Redlich et al. 2019). Few studies included dependent variable measures relating to the medium/format of the confession, such as the timing of the confession (*n* = 1; Wachi et al. 2018), the false confession rates (*n* = 8; Mindthoff et al. 2018) or the interrogator characteristics—for example, the impressions of the detective conducting the interrogation (*n* = 1; Jones and Penrod, 2018; Forrest et al. 2012). The majority of studies concerning a general/community sample were experimental in nature (*n* = 44; see Table 3).

**TABLE 3:** Matrix depicting methodologies and population type.

	General/Community	Student	Child	Offender	Professional
Experimental	44	96	9	2	6
Survey	19	30	4	47	7
Case analysis	0	0	0	11	0
Total	63	126	13	50	13

From the studies that included a general/community sample, only six studies included a vulnerable sample. Such vulnerabilities predominately related to Attention Deficit Hyperactivity Disorder (*n* = 2; Gudjonsson et al. 2007b; Gudjonsson and Young 2021; Howard 2019), a Learning Disability (*n* = 1; Gudjonsson and Young 2021; Pasala 2022), an Autism Spectrum Condition (*n* = 2; Maras and Bowler 2012; Zannella et al. 2024; North et al. 2008), Intellectual Disability (*n* = 1; O’Connell et al. 2005; Henderson and Levett 2020) and Schizophrenia (*n* = 1; Peters et al. 2012). One study included a sample with more than one type of vulnerability (Gudjonsson and Young 2021; Hall et al. 2020). In terms of the independent and dependent variables, all but one study focused on the impact of the participant type (e.g., vulnerable v control) on the psychometric test scores. O’Connell et al. (2005) explored the impact of the type of interrogation tactic on the participants psychometric test scores. Out of these six studies, only one study was experimental in nature (O’Connell et al. 2005); the other five used a survey methodology.

#### 3.3.2 Student Sample

The majority of studies utilised a student sample (*n* = 126; Alceste et al. 2020a; Barnes et al. 2023; Blair 2007; Cole et al. 2013; Forrest et al. 2012; Newring and O’Donohue 2008). The most commonly manipulated independent variable was participant type, for example, the impact of gender, or race, or age as well as the impact of the participant vulnerabilities (*n* = 102; Dierenfeldt et al. 2020; Drake et al.^2017^, ^2015^, 2015b; Gudjonsson and Sigurdsson 2010; Newring and O’Donohue 2008). Other independent variables commonly manipulated were confession veracity, for example, whether the confession was true or false (*n* = 28, Geven et al. 2020; Gudjonsson et al. 2004; Honts et al. 2013), interrogation tactics, for example, the impact of false evidence ploys or the impact of bluffing (*n* = 28; Barnes et al. 2023; Forrest et al. 2012; Swanner and Beike 2010; Wilford and Wells 2018), the presence of a confession, for example, whether a confession was present or not (*n* = 17; Appleby et al. 2011; Wojciechowski et al. 2018), and the source of the confession/evidence, for example, the impact of expert testimony or presence of an expert or how the confession was obtained such as eyewitness misidentification or a false confession (*n* = 17; Blandon‐Gitlin et al. 2011; Zannella et al. 2024). However, this sample group was rarely used in studies that included independent variables such as confession contamination warning, for example, the impact of participants being provided with instructions regarding confessions not always being true (*n* = 1; Jones and Penrod 2018), confession rehearsal, for example, the impact of rehearsing a confession once, twice or more (*n* = 1; Alceste et al. 2023), crime type/severity, for example, the impact of different types of crime such as shoplifting, drug offences and murder (*n* = 5; Najdowski et al. 2009) interrogation length for example, 1 hour v 12 h (*n* = 5; Barnes et al. 2023) or interrogation content, for example, the presence of DNA evidence or not (*n* = 2; Moffa and Platania, 2008).

The most commonly used dependent variables in studies utilising a student sample included psychometric test scores (*n* = 122; Barnes et al. 2023; Gudjonsson et al.^2006^, ^2004^, 2006a; Horselenberg et al. 2003; Steingrimsdottir et al. 2007; Villar et al. 2013), levels of perceived guilt, (*n* = 73; Alceste et al. 2020a, 2023; Appleby and Kassin 2016; Larmour et al. 2015; Serpa 2009), and false confession rates (*n* = 73; Blair 2007; Cole et al. 2013; Evans et al. 2013; Frenda et al. 2016; Perillo and Kassin 2011), as well as perceptions of the interrogation (*n* = 37; Alceste et al. 2020a; Blandon‐Gitlin et al. 2011; Mindthoff et al. 2018; Redlich et al. 2008). However, few studies included dependent variables using this sample group that focused on sentencing perceptions, for example, the likelihood of rehabilitation, the type of sentence that should be received (*n* = 8; Najdowski et al. 2009), or interrogator characteristics, for example, impressions of the detective conducting the interview (*n* = 1, Jones and Penrod 2018). Generally, the majority of studies that included a student sample were experimental in nature (*n* = 96).

#### 3.3.3 Child Sample

Overall, 13 studies utilised a child sample (Billings et al. 2006; Cleveland et al. 2018; Drake et al.^2017^, ^2015^, 2015b; Gilbert et al. 2024; Gudjonsson et al. 2021b). The most common independent variables used within this sample included participant type, for example, the age of the participant or school year group, gender, or the vulnerability of the participant (*n* = 27; Billings et al. 2006; Candel et al. 2005; Drake et al.^2017^, ^2015^, 2015b; Gudjonsson et al. 2021b; Pimentel et al. 2015). Many other independent variables were not frequently examined with a child sample. For example, the presence of a confession (*n* = 3; Evans and Lyon 2019; Gilbert et al. 2024), the impact of the source of the confession/evidence, for example, whether the confession was disclosed or not (*n* = 1; Evans and Lyon 2019), the interrogation type (*n* = 1; Billings et al. 2006), the interrogation tactics used (*n* = 6; Billings et al. 2006; Cleveland et al. 2018) and the evidence type, for example, false evidence or no evidence (*n* = 1; Redlich and Goodman 2003). Many independent variables used in other samples were not examined at all when a child sample was used—for example, the confession type, perceptions of confessions, the crime type/severity, the interrogator characteristics or the interrogation length.

The most commonly measures dependent variables in studies using a child sample included the psychometric test scores (*n* = 22; Billings et al. 2006; Drake et al.^2017^, ^2015^, 2015b; Gilbert et al. 2024; Gudjonsson and Henry 2003; Larmour et al. 2015), followed by the false confession rates (*n* = 8; Candel et al. 2005; Drake et al.^2017^, ^2015^, 2015b; Evans and Lyon 2019; Paton et al. 2018). Please note, a number of studies included more than one dependent variable. Few other dependent variables were frequently examined (if at all) when a child sample was used. For example, the confessor's characteristics (*n* = 0), the account details provided by the child sample, for example, the accuracy of information provided (*n* = 3; Cleveland et al. 2018), the perceptions of the interrogation, such as the level of appropriateness for each interrogation tactic used (*n* = 2; Hall et al. 2020) and confession knowledge (*n* = 4; Redlich and Shteynberg 2016). The majority of studies using a child sample were conducted using experimental methods (*n* = 9; Billings et al. 2006; Candel et al. 2005; Cleveland et al. 2018; Pimentel et al. 2015) rather than survey methods (*n* = 4; Drake et al.^2017^, ^2015^, 2015b; Gudjonsson et al. 2009, 2021b) and case analysis (*n* = 0; although please note, that the offender sample below includes juvenile offender groups).

From the studies that included a child sample, only two studies included children with additional vulnerabilities. Both studies utilised children with a Learning Disability that focused on the impact of the participant type (e.g., vulnerable v control) on the psychometric test scores and utilised a survey methodology (Giostra and Vagni 2024; Gudjonsson and Henry 2003).

#### 3.3.4 Offender Sample

Overall, 50 studies utilised an offender sample within the SSA and includes papers that drew on samples from the National Registry of Exonerations (Bettens and Redlich 2024; Davis et al. 2005; Frumkin et al. 2012; Gudjonsson et al. 2008, 2021; Lavoie et al. 2023; Pearse et al. 1998; Redlich et al. 2011; Sigurdsson and Gudjonsson1996a, 1996b). The most commonly used independent variables included were the participant type, for example, gender, race and age, as well as the type of vulnerability the participant may have such as a Learning Disability (*n* = 98; Arndorfer et al. 2015; Bettens et al. 2024; Bettens and Redlich 2024; Goldstein et al. 2003; Gudjonsson et al.2021a, 2008a; Iliya et al. 2022; Richardson et al. 1995), and the presence of a confession (*n* = 13; Pearse et al. 1998; Redlich et al. 2010; Richardson et al. 2014; Scherr and Normile 2022; Sigurdsson and Gudjonsson 1996a). Other independent variables were infrequently examined (if at all) in studies using this population. For example, the source of the confession/evidence (*n* = 0), the confession motivation, for example, the type of leniency promised or the reason for the false confession (*n* = 2; Deslauriers‐Varin et al. 2011; Redlich et al. 2019), the confession type for example, coerced‐compliant or coerced‐internalised (*n* = 1; Sigurdsson and Gudjonsson 1996b), the confession medium (*n* = 0), the crime type/severity (*n* = 4; Bettens and Redlich 2024; Lavoie et al. 2023; Richardson et al. 2014), the interrogator characteristics for example, the race of the primary interrogator (*n* = 1; Cleary 2014), the interrogation tactics (*n* = 6; Deslauriers‐Varin 2022; Pearse et al. 1998) nor the evidence type, for example, DNA present or not (*n* = 2; Scherr and Normile 2022).

The most commonly explored dependent variables in studies that used an offender sample included the confessor's characteristics such as the self‐reported likelihood to falsely confess (*n* = 76, Bettens et al. 2024; Cleary 2014; Deslauriers‐Varin 2022; Feld 2013; Gubi‐Kelm et al. 2020; Haney‐Caron et al. 2018; Iliya et al. 2022; Jordan 2021; Okoka and Kheswa 2024; Viljoen et al., 2005) and the psychometric scores (*n* = 78; Frumkin et al. 2012; Goldstein et al. 2003; Gudjonsson and Sigurdsson^1999, 2010^; Pearse et al. 1998; Redlich et al. 2011). Other dependent variables were rarely explored including the impact of the evidence (*n* = 2; Bettens and Redlich 2024), sentencing perceptions, (*n* = 0) and perceptions of interrogator characteristics (*n* = 0). The majority of the studies used a survey methodology (*n* = 47; Deslauriers‐Varin 2022; Gudjonsson 1990; Gudjonsson and Henry 2003; Gudjonsson et al. 2008, 2010a, 2021; Malloy et al. 2014; Pearse et al. 1998; Sehwail et al. 2019) and a case analysis (*n* = 11; Bettens and Redlich 2024; Cleary 2014; Davis et al. 2005; Feld 2013; Hagsand et al. 2022 2023).

Within the studies that included an offender sample, less than a third (*n* = 15) made an explicit distinction between adult and juvenile offenders. Furthermore, the studies within this sample type tended to focus on a male sample regardless of whether the participants were juvenile offenders (Arndorfer et al. 2015) or adult offenders (Davis et al. 2005). Very few studies made the distinction between male and female offenders with only four studies comparing differences in female and male juvenile offenders (Haney‐Caron et al. 2018; Mogavero 2020; Sigurdsson and Gudjonsson 1996a; Viljoen et al. 2005).

#### 3.3.5 Professional Sample

In total, 13 papers utilised a professional sample. The most commonly examined independent variables were training in confessions, for example, whether the professional had received any training and/or the type of training received (*n* = 18; Cleary and Warner 2016; Kassin et al. 2005, 2007), and the impact of participant type, for example, professionals such as defence attorneys v general population, differences in gender (*n* = 10; Appleby and McCartin 2019; Cleary and Warner 2016; Gudjonsson et al. 2000; Kassin et al. 2005, 2018). Less frequently explored independent variables included the impact of interrogation tactics such as whether verbal feedback was positive or negative (*n* = 2; Tata and Gudjonsson 1990).

The most commonly explored dependent variables was perceived guilt, for example, the likelihood the defendant committed the crime (*n* = 6; Brimbal et al. 2024; Levine et al. 2010), false confession rates (*n* = 4; Kassin et al. 2005), and the perceptions of the interrogation, how many guilt presumptive questions were asked for example (*n* = 4; Brimbal et al. 2024; Evans et al. 2009; Kassin et al. 2007). Other dependants variables such as the confidence in the guilt decision (*n* = 2; Kassin et al. 2005), the perceived authenticity of the confession (*n* = 1; Appleby and McCartin 2019) were infrequently examined, and other dependent variables including the impact of evidence and the account details were not examined at all within a professional sample.

The methodology was almost evenly split between experimental methods (*n* = 6; Brimbal et al. 2024; Kassin et al. 2005; Levine et al. 2010; Tata and Gudjonsson 1990) and a survey design (*n* = 7; Cleary and Warner 2016; Evans et al. 2009; Kassin et al. 2018). See Table 3.

### 3.4 Location Representativeness

**FIGURE 1:**
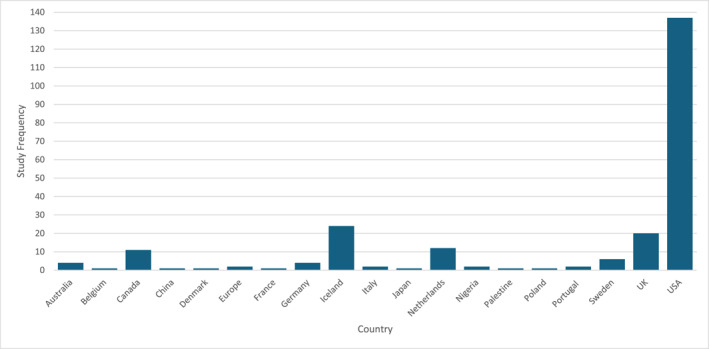
Location representativeness of studies conducted with all populations and methodologies.

Studies included in the SSA were conducted across 19 different countries around the world (see Figure 1). Studies were largely conducted in the United States of America (*n* = 137; Guyll et al. 2013; Mindthoff et al. 2020; Scherr et al. 2018; Webb et al. 2020; Woody and Forrest 2009), Iceland (*n* = 24; Pires et al. 2014; Sigurdsson and Gudjonsson 1996a, 1996b, 1997, 2001) and the United Kingdom (*n* = 20; Gudjonsson et al. 2007b; Maras and Bowler 2012; North et al. 2008; Pearse et al. 1998; Richardson et al. 1995; Tata and Gudjonsson 1990) (see Figure 1; please note, some studies were conducted in more than one country).

Experimental methods were conducted more in the United States of America (*n* = 97; Alceste et al. 2019; Henderson and Levett 2018, 2020; Redlich and Goodman 2003) than survey methods (*n* = 33; Cleary and Warner 2016; Redlich et al. 2011; Viljoen et al. 2005). This was also the case in Canada. However, many other countries conducted more survey style methodologies. For example, in the United Kingdom, survey methodologies were present in 16 studies (Drake 2010; Gilbert et al. 2024; Maras and Bowler 2012) compared to four studies using an experimental method (Nash and Wade 2009; Paton et al. 2018). This was also the case in Germany. A number of countries have not conducted any experimental work exploring confessions, for example, Iceland, Italy and Portugal, but have conducted studies utilising a survey method (Drake et al. 2017; Gudjonsson et al. 2021b; Pires et al. 2014). Studies exploring confessions have only been conducted minimal times (regardless of methodology) in a number of countries such as Belgium (Peters et al. 2012), China (Oeberst and Wu 2015), Japan (Wachi et al. 2018) and Poland (Wojciechowski et al. 2018; see Table 4).

**TABLE 4:** Matrix depicting location representativeness across methodologies and population type.

	Australia	Belgium	Canada	China	Denmark	Europe	France	Germany	Iceland	Italy	Japan	Netherlands	Nigeria	Palestine	Poland	Portugal	Sweden	UK	USA	Total
Methodology																				
Experimental	4	0	6	0	0	0	1	1	0	0	1	12	0	0	1	0	3	4	97	129
Survey	0	1	4	1	1	2	0	4	24	2	0	0	2	1	0	2	0	16	33	103
Case/Interrogation analysis	0	0	1	0	0	0	0	0	0	0	0	0	0	0	0	0	3	0	7	11
Population																				
General/Community	0	1	4	1	0	0	0	2	0	0	1	5	0	0	0	1	0	9	39	63
Student	4	0	3	1	1	2	1	3	16	0	0	11	0	0	1	0	1	5	77	126
Child	0	0	0	0	0	0	0	0	0	2	0	1	0	0	0	0	0	2	8	13
Offender	0	0	3	0	0	0	0	2	9	0	0	0	2	1	0	2	5	5	21	50
Professional	0	0	1	0	0	0	0	0	0	0	0	0	0	0	0	0	0	2	10	13

The populations that were used within all studies varied by country. For example, the United States of America tended to use a student sample (*n* = 77; Appleby et al., 2011; Cole et al., 2013; Evans et al., 2024; Woody and Forrest, 2009; Evans et al. 2009) more frequently than a general/community sample (*n* = 39; Alceste et al. 2019; Bernhard and Miller 2018). This was also the case for Iceland; the studies included in the SSA from Iceland tended to use a student sample (*n* = 16; Drake et al.,^2017^, ^2015^, 2015b; Gudjonsson et al. 2004) or an offender sample (*n* = 9; Gudjonsson et al. 2008, 2010; Sigurdsson and Gudjonsson 2001). No studies included a general/community sample in Iceland. The studies conducted in the United Kingdom, however, focused on all sample types. The majority were conducted using a general/community population (*n* = 9; Drake et al. 2008; Gudjonsson and Clare 1995; North et al. 2008), but a similar number of studies were conducted using a student and offender population (*n* = 5 respectively; Nash and Wade 2009; Pearse et al. 1998). A child population and a professional population were used each in two studies. There were very few studies conducted in other countries that focused on each sample group (see Table 4).

## 4 Discussion

The aim of the research was to examine the adequacy and concentration of studies on false confessions in order to systematically determine where (if any) significant gaps of knowledge remain. Using a Study Space Analysis (SSA), it was found that a variety of independent and dependent variables have been examined, but that there remain some issues that have been neglected or have received little replication. In addition, whilst there has been a vast array of experimental work conducted, further focus on real‐life cases is needed and there are some key populations missing from the literature. Finally, the SSA determined that the majority of all research work conducted was in the United States of America (USA). Each area will be explored in turn.

### 4.1 Independent and Dependent Variables

The SSA identified a number of independent and dependent variables that were frequently examined against each other. However, there were also many gaps identified. For example, the impact of the source of the confession/evidence, for example, a confession or a false incriminating witness, was rarely explored in relation to the interviewee's (participants used in an experimental nature) or the confessor's (real‐life exonerees) characteristics—that is, the source of the confession/evidence was rarely examined against the perceived pressure to confess, nor the perceptions or consequences of confessing, nor their level of understanding of the criminal justice process or other individual characteristics such as education levels, offending history, or previous experience of completing interrogations. Research has documented that eyewitness identification is a compelling piece of evidence although it is frequently identified as a contributing factor to wrongful convictions (National Registry of Exonerations 2022), but little work has examined the impact of evidence type on confession decision‐making. Given the limited attention this has been given, there remains little understanding as to the interaction between the source of the confession/evidence and the individual characteristics that may result in a false confession. In addition, the type of false confession obtained (e.g., coerced v voluntary) was also rarely examined in relation to interviewee or confessor characteristics—that is, there remains little understanding as to what type of confession may be elicited from what type of individual based on characteristics detailed above, for example, perceived pressure to confess, consequences of confessing etc. Whilst this may be due to time constraints, this is concerning given that it is theorised that there are a number of risk factors that may increase the likelihood of providing a false confession (Gudjonsson 2018). In addition, some individuals may falsely confess even if they have not been coerced (Hughes and King 2024). Given the little work that has explored this, there remains a lack of knowledge in understanding the interaction between personal factors (confessors characteristics, type of confession) and the situational factors (impact of evidence type) that may place individuals at risk of falsely confessing.

A plethora of work has been conducted on the impact of the suspect/defendant type used in vignettes in experimental work on perceived levels of guilt, for example, if the suspect/defendant is guilty based upon characteristics such as race and level of vulnerability. However, there has been little work that has explored the impact of the suspect/defendant type against the confidence in the guilt decision made, for example, how confident the individual is in their verdict of guilt. This is concerning given that other research work has documented that despite being no more accurate than a layperson in identifying a true from a false confession, police officers, for example, were more confident in their judgements (Kassin et al. 2005). Utilising a female suspect/defendant within the vignettes, an area i.e. relatively underexplored, may impact upon perceived guilt and confidence ratings of guilt. Given that the first stage of the Reid Interrogation Technique is the Behaviour Analysis Interview in which the suspect is judged to be innocent or guilty, developing understanding as to what can guide guilt decisions and confidence in those guilt decisions is crucial. This is also of particular importance given the jury decision‐making process in England and Wales, and other countries around the world. We must be able to understand what can impact upon a jury decision‐making. However, being able to do so is not an easy task. In England and Wales, e.g., researchers and the public are not allowed to observe the jury coming to their decision in real‐life cases and so in order to understand this decision‐making, there is often a reliance on experimental methods which can be time‐consuming and difficult to conduct. Other independent variables that have rarely been examined against perceived guilt and confidence in guilt decisions include the type of instruction provided to juries that may influence their decision‐making. Given that juries are likely to convict an individual based on confession evidence alone, even with the knowledge that the confession obtained was coerced (Appleby and Kassin 2016), more research needs to explore if and how different types of jury instruction may have an impact. Finally, the SSA identified that the independent variable relating to training in confessions was infrequently examined against perceived guilt, and the confidence in guilt decisions. Whilst it is positive that training is being delivered, the impact of it is not understood. Research has documented the effective impact that education and training may have on informing the general public and professionals about confessions (Stewart et al. 2018). Research could explore the impact that training in false confessions may have on the likelihood to convict an individual who provided a coerced false confession and could have huge implications for the justice system.

### 4.2 Ecological Validity

The majority of the research work identified in the SSA tended to use experimental methods. Scholars have highlighted the importance of using experimental work as analogues for real‐world practice, especially if the materials and procedures used are based on real‐world, for example, the type of interrogation tactics that may be used, and elicit meaningful physiological and psychological changes in the participants (Guyll et al. 2013). Furthermore, experimental studies allow for false confessions to be explored in controlled situations whereby variables can be controlled, and researchers have access to the ground truth (Stewart et al. 2018.) However, by their very nature, experimental studies are not able to exactly mirror real‐world practice, and whilst they allow for stronger internal validity, ecological validity, overall, is reduced.

A large number of experimental studies included in the SSA tended to use a well‐known Alt‐Delete computer crash paradigm (Kassin and Kiechel 1996) or the cheat test paradigm (Russano et al. 2005). Both paradigms have been repeatedly used in further experimental work (Forrest et al. 2006; Houston et al. 2014; Klaver et al. 2008). Whilst there are some similarities in the use of these paradigms to real‐life interrogations, for example, minimisation techniques in the Alt‐Delete paradigm (Stewart et al. 2018), the consequences for confessing are relatively mild. Thus, their findings may not represent the decision‐making process an individual experiences in an interview or interrogation room. In addition, a recent meta‐analysis found that false confessions were more likely in typing studies (such as the Alt‐Delete paradigm) than in cheating studies (Stewart et al. 2018). Whilst this warrants further attention, the difficulties of observing real life confessions as they occur in the police interview or interrogation must be noted. That said, it must also be acknowledged that scholars are becoming more aware of the need for ecological validity and there is an increase in trying to adopt ecological valid methods into experimental work.

In addition to the experimental work conducted, a large number of studies adopted a survey method whereby correlations were drawn between participant characteristics for example, for example, psychometric scores and the likelihood for false confession. The assumption of this approach is that individual differences play a role in the variability in false confession rates (Gudjonsson et al. 2010). These types of studies were particularly prevalent among student and offender populations respectively.

In contrast, a total of 11 studies focused on analysing real‐life cases or interrogations. Scholars have highlighted that most published work focuses on experimental methods (Deslauriers‐Varin, 2022) and indeed, this has been the finding of the current paper. However, case studies remain invaluable given the insight they can provide from their rich data and high levels of ecological validity (Stewart et al., 2018). That said, given the complexity of the data and in particular, the interactions between factors that may cause a false confession, there remain issues with generalisability (Stewart et al., 2018). Consequently, scholars have called for collaboration between those working within the justice system and academic researchers to increase the realism of experimental research methods. It is proposed that research encompassing both experimental methods and case studies make important contributions to the understanding of confessions and that collaboration between practitioners and academics will strengthen some of the aforementioned pitfalls.

### 4.3 Population

The work included in the SSA identified five main types of population used within the research work: (i) general/community, (ii) student, (iii) child, (iv) offender, and (iv) professional. Whilst there were some areas that were well explored within each population type, the SSA highlighted a number of gaps. For example, in the general/community sample, minimal studies explored independent variables pertaining to jury instructions, confession details, confession medium, perceptions of confessions or interrogation content. Furthermore, dependent variables relating to the timing of the confession, false confession rates or interrogator characteristics were also rarely explored. Of note, only six studies using this type of sample, used a vulnerable sample. This is not overly surprising given the difficulties in accessing this sample type and the ethical framework in which psychological research must be conducted. That said, research has frequently reported that vulnerable individuals, such as those with a learning disability or mental health condition, are at a heightened risk of providing a false confession (Gudjonsson 2018) and so further attempts must be made to include this sample type in future work.

The majority of studies included in the SSA utilised a student sample. However, this sample group was rarely used in studies that focused on independent variables such as the presence of a confession contamination warning for example, the impact of participants being provided with instructions regarding confessions not always being true, or confession rehearsal. Given that the majority of research work included in the current SSA used a student sample, and that research has documented that observers were more likely to believe false practiced statements compared to once told statements, it is concerning that research has not focused on these elements with this sample group. Also of note, research conducted with this sample group rarely focused on variables relating to different types of crimes used in vignettes for example, nor the impact of interrogation length or content, despite research documenting significant differences in the length of an interrogation that has elicited a false confession (Drizin and Leo 2004). This sample were also rarely used in studies that focused on sentencing perceptions or interrogator characteristics. This is surprising given the ease in accessibility to this type of sample. However, scholars have noted that this population can be over‐relied upon which is concerning given their limited experience of the justice system (Welner et al. 2024).

The SSA identified 13 studies that utilised a child sample. Predominately, these studies tended to explore independent variables such as differences in the age of the child, their grades in school, and whether they had been maltreated or not and dependent variables such as their psychometric test scores, and their false confession rates. Many variables were infrequently or not examined including the interrogation type they experienced, the type of evidence that was provided against them or the type of interrogator they had, and the type of account details they provided such as the accuracy of information they provided. This is concerning given that children are at a heightened risk of providing false confessions (Billings et al. 2006; Crane et al. 2016). Such likelihood to provide a false confession is exacerbated when the child has other vulnerabilities, for example, a Learning Disability. Only two studies included in the SSA included children with a Learning Disability (Giostra and Vagni 2024; Gudjonsson and Henry 2003)—both focused on the difference in psychometric test scores between the vulnerable child compared to a control. The potential risks associated with this population type are well documented in popular TV series (e.g., Brendan Dassey in ‘Making a Murderer’). It is concerning that the rate of knowledge does not appear to be keeping up with the rate of juvenile crime, although access to this population can be fraught with difficulties.

The majority of research studies that used an offender sample tended to focus on variables pertaining to their characteristics, for example, young offenders v adult offenders, or number and type of negative life events. Surprisingly, variables such as confession motivation, for example, the type of leniency promised or the reason for the false confession, the confession type for example, (coerced‐compliant v coerced‐internalised), the type of tactics used in the interrogation and the evidence type were rarely examined. This is concerning given that research has indicated that techniques from accusatorial and information gathering approaches can predict a suspects' self‐reported confession and disclosure decisions (Bettens 2024). The need for replication studies with suspects/defendants who have been interrogated and prosecuted have been called for (Bettens 2024). Furthermore, the majority of studies using this population type tended to focus on a male sample—very few studies made the distinction between male and female offenders with only four studies explicitly comparing differences between the two (Haney‐Caron et al. 2018; Mogavero 2020; Sigurdsson and Gudjonsson 1996a; Viljoen et al. 2005). This warrants much more attention given that research has shown there to be gender differences in false confessions with a higher proportion of females providing false confessions than males (Jones 2011) although the limited results available appear mixed. Whilst the female offender population is significantly lower than the male offender population and so may be a reason for the limited academic inquiry, the complexities and challenges faced by females in prison (e.g., disproportionate level of self‐harm, impact on children) warrant the need for further work.

The last type of sample used within the studies included in the SSA focused on a professional sample. Most commonly explored variables include training in confessions, for example, whether the professional was trained in the Reid technique, or whether they were trained or untrained. Despite this, gaps in knowledge using this sample group were identified. For example, little research focused on variables including impact of evidence, or confidence in guilt decisions. Given that research has identified how legal professionals can adopt confirmation bias which can lead to guilt confirming evidence and the disregarding of exoneration information, further research needs to focus on the factors that can lead to this. This has implications for all those involved in the justice system, particularly those who have provided the confession.

### 4.4 Location

The studies included in the SSA were conducted across 19 different countries. Whilst this is encouraging, especially given that the USA, who still endorses the Reid Interrogation Technique, had the highest number of studies that focused on false confessions, the SSA has identified that research work focusing on false confessions is only being conducted in a small number of countries despite the problem being worldwide (Le et al. 2024). For example, in a recent review, Le et al. (2024) reported that only 10 countries in Asia published research on false confessions and wrongful convictions despite false confessions being the most common wrongful conviction cause in Asia (Chen and Chua 2010; Johnson 2015; Liang et al. 2019), with some scholars identifying that the criminal justice professionals in their country see a confession as essential to a successful prosecution (Hui and Lo 2015). Given the mindsight of such criminal justice professionals, it is understandable that false confessions are not of research importance in their respective countries. Furthermore, in the countries where the research work is being conducted, it differs in terms of methodology. The USA and Canada tend to focus more on experimental methods, whilst the United Kingdom (UK) and Germany frequently utilise survey methods. Thus, there remains strengths and weaknesses in terms of ecological validity, representativeness and generalisability. This may also impact upon current knowledge in each respective country and their subsequent policy decision‐making.

### 4.5 Limitations

Despite the vast amounts of research conducted into false confessions, the SSA has provided important insight into areas where little or no research work has been conducted at all. However, the limitations of the SSA method must be acknowledged. The SSA does not claim to be an exhaustive review of all literature (Amurun 2021). For example, the current SSA did not include theses or dissertations, and the search terms were not only limited in number but were conducted solely online. Furthermore, the search sought papers that had been written only in English; this must be acknowledged when considering which country produced the most research. This may have resulted in some relevant research being missed from the final sample (McGinn et al. 2016). That said, five large databases were explored, and the initial search terms resulted in over 1000 published pieces of work. Furthermore, unlike other review practices, the role of the SSA is not to examine the quality or validity of the findings from the research included and so it is possible that findings may not be consistent, nor reliable or valid (Waterhouse et al. 2020). However, the SSA has been used to determine the spread of research exploring false confessions in order to identify areas of little interest and where further work may be needed. This is vital given that despite the plethora of research work conducted to date, there still remains many areas for investigation.

### 4.6 Future Directions

The SSA has identified a number of areas that require further attention. Inquiry into the source of the confession/evidence type and its impact on perceived pressure to confess, perceptions relating to consequences of confessing and the overall level of understanding of the criminal justice system is needed. Another strand of research should focus on whether the suspect/defendant type impacts on confidence in verdicts of guilt. For example, are specific suspect/defendant types more likely to elicit a confident guilty verdict? Similarly, the impact of different evidence types on perceived guilt and confidence in guilt decisions should be considered, as should the impact of training or jury instructions. Utilising under‐represented samples is a key future direction; for example, those who are vulnerable, and female suspect/defendant/confessor should be considered in further research work that explore perceived guilt and confidence ratings of guilt. Work exploring false confessions across a range of different crimes and crime severity should be considered, and there needs to be an emphasis on further case analysis with particular attention paid to the experience of interrogation and tactics used, the impact of evidence presented, and the type of confession provided and the motivation for it. Replication studies of work conducted in the UK and USA could be considered for countries that are lacking research inquiry into false confessions.

### 4.7 Conclusion

False confessions are not a new phenomenon. It is well understood that a false confession can lead to a wrongful conviction; the latter is a violation of human rights and has long‐term and complex consequences for the individual involved (Garrett 2020; Le et al. 2024; Leo and Gould, 2009). The problem is that individuals tend to over believe confessions even when knowing that the confession has been obtained using coercive interrogational tactics or contradicted by other evidence (Appleby and Kassin 2016). This has implications for the justice system.

The overall aim of the SSA was to explore the existing research base concerning false confessions, in order to examine what research work has been conducted and to determine where further inquiry is needed. There has been some extensive work conducted which has undoubtedly led to the development of existing knowledge surrounding false confessions. Policymakers are required to draw upon this literature and knowledge in developing their policies; if the literature does not exist, neither can the knowledge nor the policy. Thus, there remains much more to be done.

## Ethics Statement

The author has nothing to report.

## Conflicts of Interest

The author declares no conflicts of interest.

## Data Availability

All materials and data for this analysis are available upon request from the author.
